# Doubtful Tumours of the Breast

**Published:** 1900-05-05

**Authors:** 


### DOUBTFUL TUMOURS OF THE BREAST.

The urgency with which all recent teaching has insisted upon the importance of early and complete removal in every case of cancer of the breast undoubtedly leads to a certain danger of occasionally removing breasts which are not cancerous—breasts which might have been safely treated by less heroic operative measures. Indeed, so firmly is the professional mind impressed with the idea that any tumour, not inflammatory, appearing in the breast of a woman over 40 years of age, is probably cancerous, as almost to forbid any other explanation of its nature being entertained. Yet, as was shown by Mr. Bryant in a paper read at the last meeting of the Medical Society, many cases of tumour of the breast, occurring about this age, are simple cysts, and can be quite properly treated by comparatively small and simple operations. He goes so far as to say that if one puts on one side the inflammatory cases, and those in which there can be no reasonable doubt as to their being cancerous, "and apply our argument to those alone of a doubtful nature, in which there are no collateral symptoms of cancer to support a diagnosis, it does not appear to be wrong to conclude that in every two cases one will be cystic." From a careful review of his experience, Mr. Bryant comes to the conclusion "that simple cysts are far more common than they are generally believed to be; that they are chiefly found in women during the same period of life as that in which cancer is met with; that they are mostly quite amenable to local treatment, without the sacrifice of the breast gland in which they are situated; and that there is no reason to believe that women who have these cysts are more prone to cancer than those who have them not." The treatment he adopts for these cysts is by a free incision to expose, to empty, and to remove them. Should the cyst, when exposed, present a lining membrane absolutely free from anything like papillary or other growths, its excision is not a necessity, "for when its inner surface has been destroyed by swabbing it with either pure liquid carbolic acid, a strong solution of zinc chloride, or the tincture of iodine, there is but little probability that any growth will subsequently appear." If any intracystic growth, however small, should be found the cyst must be dissected out, and if this growth is of a fleshy nature the lobe, or perhaps the whole breast, should be removed. It must not be imagined that Mr. Bryant advises that cystic cases should be let alone. They should be operated on just as much as cancers should, but the operation which they require is in very many instances of a comparatively trifling character. The importance of bearing in mind the possibility of a doubtful case being a simple cyst is all the greater in view of the very large operations now in vogue in the treatment of cancer, the only disease that such a cyst occurring at such an age is likely to be confounded with.

